# 

**Published:** 1887-11-05

**Authors:** 


### CONTENTS.

**A Call for Recruits from Clergy and People** - 89

**Words of Consolation.**—Chapter LVII. The Sufferings of Christ - 90

**The Sick, the Churches, and the Hospitals.** By Sir ANDREW CLARK, Bart., M.D., F.R.S., President of the Hospitals Association - 91

**The Doctor's Pulpit.**—Woman at Home - 91

**From Friends Across the Sea.** By our READERS' RELATIVES AND COLONIAL COUSINS - 93

**The National Pension Fund** - 94

**Clinical and Therapeutic Notes.** By C. R. ILLINGWORTH I. "For the Blood is the Life" - 95

**Notes and News.**—Royal Infirmary, Liverpool.—Nurses Hours of Work.—The Hospitals Association.—Teaching the Blind to Read in China.—Evil Effects of Regular Habits.—Hydrophobia.—Sir Andrew Clark's Scheme for Supporting Hospitals.—As Others See Us.—Mutual Friendly Aid Society.—Circular Wards in Hospitals.—St. Mary's Cottage Hospital, Northam, Southampton.—The Case of Miss Buchanan.—Miscellaneous Items, &c., &c. - 96

**Annotations.**—"Go thou and do likewise."—Intelligent Reciprocity.—Farcical Inquests - 99

**The Students' Corner** - 100

**Architects, Hospitals, and Asylums.** By HENRY C. BURDETT - 102

**East and West.** By LESLIE KEITH Chapter VI. - 104

**Amusements with Profit, for all Ages.**—For Men and Women.—The Children's Corner - 106

